# Nonlinear down-conversion in a single quantum dot

**DOI:** 10.1038/s41467-022-28993-3

**Published:** 2022-03-16

**Authors:** B. Jonas, D. Heinze, E. Schöll, P. Kallert, T. Langer, S. Krehs, A. Widhalm, K. D. Jöns, D. Reuter, S. Schumacher, A. Zrenner

**Affiliations:** 1 Paderborn University, Physics Department, Warburger Straße 100, 33098 Paderborn, Germany; 2 Paderborn University, Center for Optoelectronics and Photonics Paderborn (CeOPP), Warburger Straße 100, 33098 Paderborn, Germany; 3 Paderborn University, Institute for Photonic Quantum Systems (PhoQS), Warburger Straße 100, 33098 Paderborn, Germany; 4 Wyant College of Optical Sciences, University of Arizona, Tucson, AZ 85721 USA

**Keywords:** Single photons and quantum effects, Quantum dots, Nonlinear optics

## Abstract

Tailored nanoscale quantum light sources, matching the specific needs of use cases, are crucial building blocks for photonic quantum technologies. Several different approaches to realize solid-state quantum emitters with high performance have been pursued and different concepts for energy tuning have been established. However, the properties of the emitted photons are always defined by the individual quantum emitter and can therefore not be controlled with full flexibility. Here we introduce an all-optical nonlinear method to tailor and control the single photon emission. We demonstrate a laser-controlled down-conversion process from an excited state of a semiconductor quantum three-level system. Based on this concept, we realize energy tuning and polarization control of the single photon emission with a control-laser field. Our results mark an important step towards tailored single photon emission from a photonic quantum system based on quantum optical principles.

## Abstract (web summary)

Tailoring the properties of single photon emission is a key requirement in photonic quantum technologies. Here, the authors demonstrate frequency and polarisation control of photons emitted from quantum dots through a laser-controlled down-conversion process.

## Introduction

Today’s quantum dot (QD) research and applications employ resonant excitation schemes, which give access to functionalities known from two-level systems. This is the basis for coherent state control and manipulation of excitonic states, which are important for photonic quantum technologies^1^. Rabi rotations are the standard technique for the excitation of single photon emitters^2–6^. This approach has proven to be the best scheme to realize deterministic sources of indistinguishable photons^7^, which are required for the exploitation of single photon nonlinearities, based on the Hong-Ou-Mandel effect (HOM) in photonic quantum computing and simulation^8,9^. For the generation of polarization entangled photon pairs, this scheme was extended to the resonant two-photon excitation (TPE) of the biexciton state $|B\rangle$. In the schemes described so far the spectral and polarization properties of the emitted photons are defined by properties of the quantum states, which are involved in the emission process. The quantum states can also be fine-tuned by applying an electric field^10,11^, a magnetic field^12^, or strain^13–16^. Also photonic methods have been employed in the past to control the emission of single quantum systems. A prominent example is the Λ-system, where Raman-like transitions via a virtual state lead to single photon emission that can be controlled by the spectral properties of the driving laser field^17^. In a different approach, a cavity has been used to steer the biexciton decay into a degenerate, spontaneous two-photon decay path^18^.

In the present contribution we introduce and demonstrate a concept, which enables the control of single photon emission from an individual semiconductor QD by a nonlinear down-conversion process. Starting from a biexciton state $|B\rangle$, a tunable control-laser field defines a virtual state for a stimulated down-conversion (SDC). Related concepts, which are reminiscent to stimulated Raman scattering^19^, have been studied already in atomic systems^20–22^.

## Results

**Fig. 1:**
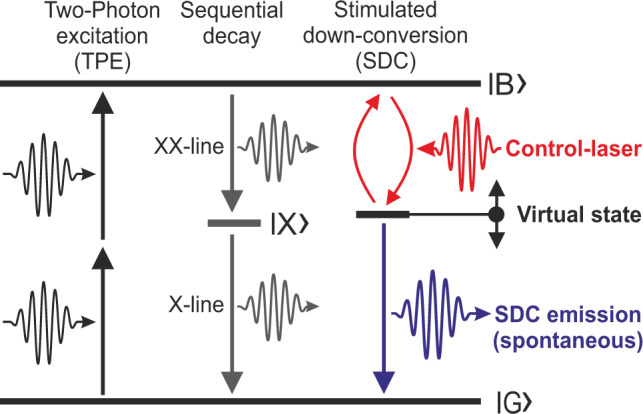
Excitation and down-conversion scheme. Two-photon excitation from the ground state $|G\rangle$ to the biexciton state $|B\rangle$ and stimulated down-conversion (SDC) to a virtual state by a control laser-field. The energy of the spontaneous SDC emission is defined by the energies of the state $|B\rangle$ and the control-laser energy and detuned from the cascaded decay via the exciton state $|X\rangle$.

**Fig. 2:**
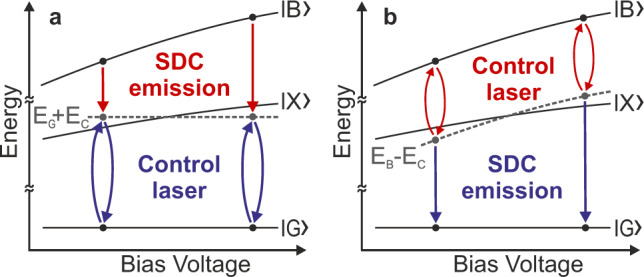
Stark shift behavior of the SDC-emission. **a** A control-laser energy higher than the TPE laser energy results in SDC-emission from $|B\rangle$ to the virtual state at *E* = *E*_G_ + *E*_C_. **b** A control-laser energy lower than the TPE laser energy results in SDC emission from the virtual state at *E* = *E*_B_ − *E*_C_ to $|G\rangle$. In both cases the Stark-shift of the SDC emission is given by the Stark-shift of the $|B\rangle$ state.

The SDC process in a single QD is schematically outlined in Fig. 1. In an initial step, a resonant TPE of the biexciton state $|B\rangle$ with energy *E*_B_ is performed. By driving the system with a tunable, off-resonant control-laser field, a virtual state for the SDC process is defined. From there, spontaneous emission to the ground state $|G\rangle$ leads to optically controlled single photon emission. The SDC-emission energy *E*_SDC_ is therefore correlated with the control-laser energy *E*_C_ and the simple energy-conservation *E*_B_ = *E*_C_ + *E*_SDC_ holds. Please note that in Fig. 1 we only show the scenario where the virtual state is defined relative to $|B\rangle$. In an equivalent scenario the virtual state can be defined relative to $|G\rangle$ (see Fig. 2).

The initial biexciton state is composed of two electrons and two holes, each with antiparallel spins, in the s-shell of the QD. Therefore, the initial state $|B\rangle$ and the final state $|G\rangle$ are both spin-zero states. Consequently, the spins of the involved photons (control and SDC), must add up to zero. Hence, the energy and polarization of the single photon emission can be controlled by the energy and polarization of the classical control-laser field. This optical control of tailored single photon emission has been intensively studied in theory, with a special focus on timing and selection rules^23^, spectral properties of the resulting emission^24^, and methodological parallels to the description of Λ- and V-type systems^25^.

In existing work on the single QD spectroscopy of dressed excitonic states the spectral features and tuning behavior of the Autler-Townes splitting was investigated under near resonant conditions^26,27^. The underlying physics is closely related to our experiments and provides valuable insight into the nature of the down-conversion process studied here. In our work we step forward to an extended detuning regime, which we describe as stimulated down-conversion. This allows for a unified description of spectral tuning and polarization control of single photon emission in a single intuitive model.

It is expected that resonance enhancement of the efficiency of the SDC can be observed when the laser induced virtual state is near the $|X\rangle$-state. Based on this consideration and the conservation of energy, there are two favorable scenarios for the demonstration of SDC. Here, the control-laser is either tuned close to the X- or the XX-line, resulting in SDC emission in the vicinity of the XX- or X-line respectively (see Fig. 2). To ensure an unambiguous identification of the down-converted emission we make use of the Stark effect tuning characteristics of the exciton states in the QD. In Fig. 2 we schematically show the tuning characteristics of the single exciton state $|X\rangle$ and the biexciton state $|B\rangle$. The latter has a Stark shift roughly twice as large as the shift of the $|X\rangle$ state. If we switch on a control-laser field with a photon energy *E*_C_, there are two equivalent options for the formation of a virtual state. In Fig. 2a, we have illustrated a scenario, where a virtual state at *E* = *E*_G_ + *E*_C_ forms the final state for a spontaneous SDC decay from the biexciton state $|B\rangle$. An alternative scenario is illustrated in Fig. 2b, where a virtual state at *E* = *E*_B_ − *E*_C_ forms the initial state for a spontaneous SDC decay to the ground state |*G*〉. For both scenarios the SDC decay with energy *E*_SDC_ appears with a Stark shift, which corresponds to the shift of the biexciton state $|B\rangle$. This behavior is a direct consequence of the expected conservation of energy: *E*_C_ + *E*_SDC_ = *E*_B_. In this work we use phonon-assisted two-photon excitation^28,29^. This allows for biexciton generation that is robust against detuning. Under this condition the SDC-emission can be identified by its unique Stark shift fingerprint, which deviates from the behavior of the allowed and competing X- and XX-lines. Following this strategy, we analyzed the down-conversion process in bias voltage dependent experiments.

**Fig. 3:**
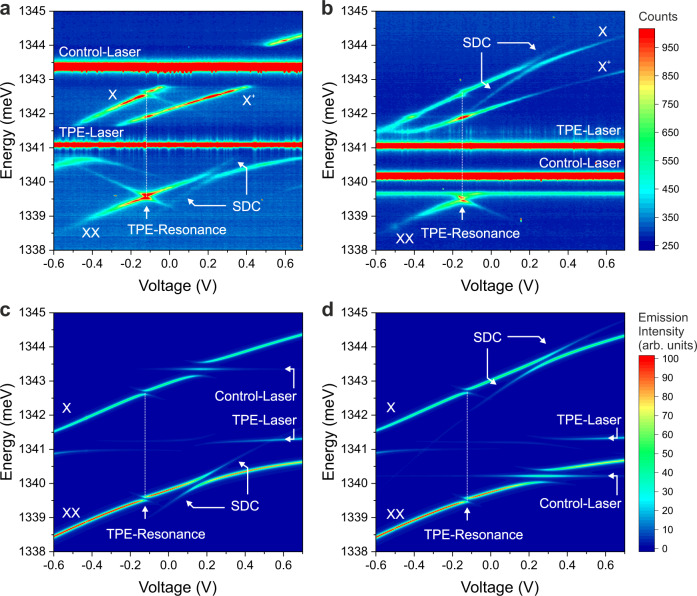
Stimulated down-conversion (SDC) in a single quantum dot. **a** Experiment with blue-shifted control laser: The SDC emission appears on the low-energy side with the expected Stark shift and an avoided crossing with the XX-line (*P*_TPE_ = 10 mW, *P*_Control_ = 1.5 mW, *T*_int_ = 2 s, ħΩ_0_ = 0.198 meV). **b** Experiment with red-shifted control laser: The SDC emission appears on the high-energy side with the expected Stark shift and an avoided crossing with the X-line (ħΩ_0_ =  0.189 meV). **c** Theoretical results for case **a**, taking into account the experimental conditions. **d** Theoretical results for case (**b**).

In Fig. 3 we present our results, which conclusively demonstrate laser-controlled down-conversion in a single QD. For this we applied control-laser energies above (Fig. 3a) and below (Fig. 3b) the TPE laser energy. For both cases we observe the predicted down-conversion process (labeled SDC), in each case on the complementary energy side and also with the expected magnitude of the Stark shift, given by the tuning behavior of the biexciton state $|B\rangle$ (sum of the slopes of the X- and XX-lines).

**Fig. 4:**
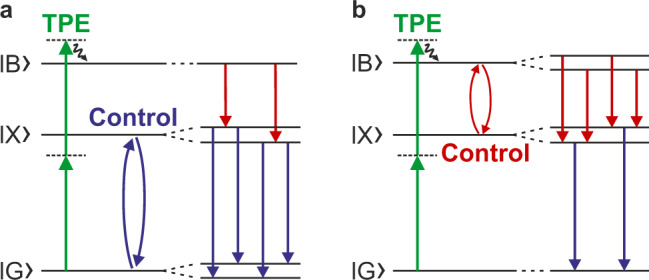
Energy levels for resonant control laser. **a** The control laser drives the $|X\rangle$–$|B\rangle$-transition, creating dressed states and forming a Mollow triplet. The |*B*〉–|*X*〉-transition is split in two lines. **b** The control laser drives the |*B*〉–|*X*〉-transition, creating dressed states and a Mollow triplet. The|*B*〉–|*X*〉-transition is split in two lines.

When the X-line is tuned into resonance with the control-laser (see Fig. 3a for a bias voltage of *V*_Bias_ = 0.2 V), the single exciton state $|X\rangle$ and ground state $|G\rangle$ are split (dressed) and form a Mollow-triplet^27^ (see level scheme in Fig. 4a). For the experimental conditions (*P*_TPE_ = 10 mW, *P*_Control_ = 1.5 mW) we determined a Rabi energy of $\hslash {\Omega }_{0}=0.198\,{{{{{\rm{meV}}}}}}({\Omega }_{0}/2\pi =47.9\,{{{{{{\rm{GHz}}}}}}})$ from the splitting of the XX-line. This interpretation is supported by the comparison with our theoretical results shown in Fig. 3c. Details on the theoretical approach are given below and in the Methods Section. In this case the SDC process takes place between the $|B\rangle$ and a dressed $|X\rangle$ state (split) and the XX-line shows an avoided crossing with the SDC-emission. The comparison of our experimental results with the simulation shown in Fig. 3c, shows very convincing agreement and all features can be explained with our model.

We note that notch-filters are used in the experiment to suppress stray light from the TPE- and control-laser. In a narrow range around their center energies (about ±0.4 meV) the QD emission is therefore blocked.

The theoretical results shown in Fig. 3c, d are based on a density matrix description of the system including the relevant electronic configurations $|G\rangle$, $|X\rangle$, and $|B\rangle$ and classical coherent light fields. The von-Neumann equation of motion for the system density operator is solved in matrix representation including pure dephasing and radiative losses guided by the experimentally determined parameters. An additional incoherent source is included to enable biexciton excitation for the case when the two-photon resonance condition is not met by the laser source (in experiment enabled by phonon-assisted excitation). Emission spectra are obtained from the time-integrated Fourier transform of the first order correlation function g^(1)^(t,τ). Details are given in the “Methods” section.

For a control-laser energy below the TPE laser energy (Fig. 3b), the SDC-emission and the avoided crossing is experimentally observed on the X-line at *V*_Bias_ = 0.2 V. From the splitting of the X-line we determined a Rabi energy of $\hslash {\Omega }_{0}=0.189\,{{{{{\rm{meV}}}}}}({\Omega }_{0}/2\pi =45.7\,{{{{{{\rm{GHz}}}}}}})$ (see Supplementary Note 4 for this and further details of the SDC emission). In analogy to the previous case, the Mollow-triplet appears now on the XX-line as observed in the theoretical results in Fig. 3d. The corresponding level scheme is shown in Fig. 4b.

**Fig. 5:**
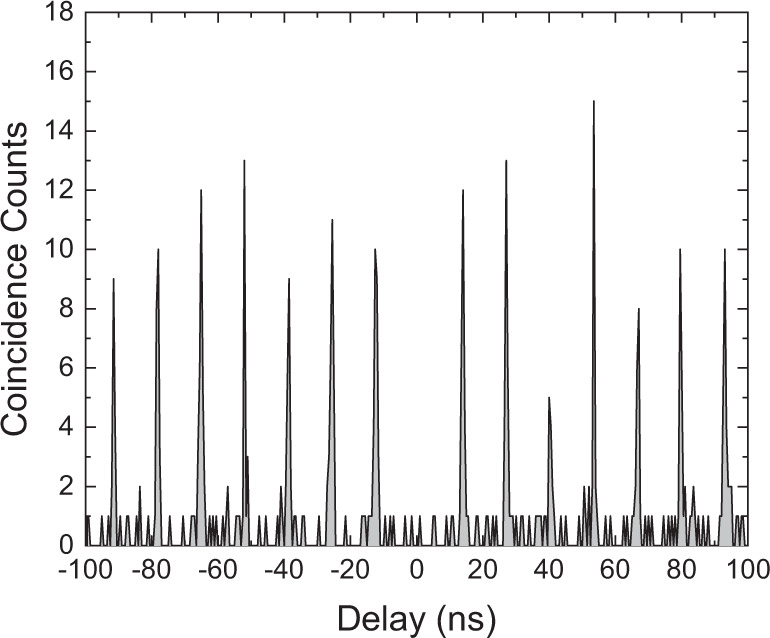
g^(2)^-measurement of SDC emission. Histogram of the pulsed correlation measurement. Because of the weak SDC intensity an integration time of 118 h has been used. The central peak is virtually missing (g^(2)^(*t* = 0) < 0.1), indicating true single photon emission.

To verify, that the SDC is in fact a single photon emission process, we performed an Hanbury Brown-Twiss experiment. In this experiment the biexciton state was prepared by resonant two-photon excitation, using a ps laser source with a pulse area of π. The control-laser was operated in cw mode to ensure a spectrally sharp SDC emission. The control-laser was tuned to an energy of 0.255 meV below the XX-emission, resulting in an SDC emission at 0.469 meV above the X-emission. The SDC signal was filtered using a combination of a bandpass- and notch-filters, sent to a 50:50 fiber-beamsplitter with two superconducting single photon detectors at the output. The resulting histogram obtained from a time tagger is shown in Fig. 5. As a result of the pulsed state preparation the SDC emission appears also pulsed. The diagram shows clear anti-bunching at zero time-delay, proving the single photon character of the SDC emission.

As briefly introduced above, the nonlinear down-conversion can be used as a tool to tailor the polarization of the emitted photons^23^. This mechanism is based on the optical selection rules for a two-photon transition between two states with a total angular momentum of zero. In our experiment these two states are represented by the biexciton state and the ground state. This means that the photon spin of the control-laser and SDC-photon must add up to zero, like in a two-photon absorption process^30^. In case of a linearly polarized control-laser, this means that the SDC-emission must have the same linear polarization, while in case of a circularly polarized control-laser the SDC must have the opposite circular polarization to ensure the conservation of the total angular momentum in the system. This concept can be generalized to arbitrary polarizations, using Stokes vectors. The Stokes parameter ${s}_{3}$ represents the ellipticity of the polarization state. This parameter has opposite signs for control and SDC, while all other parameters stay constant:

1 $${s}_{1,{{{{{{\rm{SDC}}}}}}}}={s}_{1,{{{{{{\rm{Control}}}}}}}}$$

2 $${s}_{2,{{{{{{\rm{SDC}}}}}}}}={s}_{2,{{{{{{\rm{Control}}}}}}}}$$

3 $${s}_{3,{{{{{{\rm{SDC}}}}}}}}=-{s}_{3,{{{{{{\rm{Control}}}}}}}}$$

In order to demonstrate this prediction, we performed a series of experiments with a systematic variation of the control-laser polarization and a polarization analysis of the SDC-emission. Thereby, we considered the two cases of linear and circular control polarizations.

**Fig. 6:**
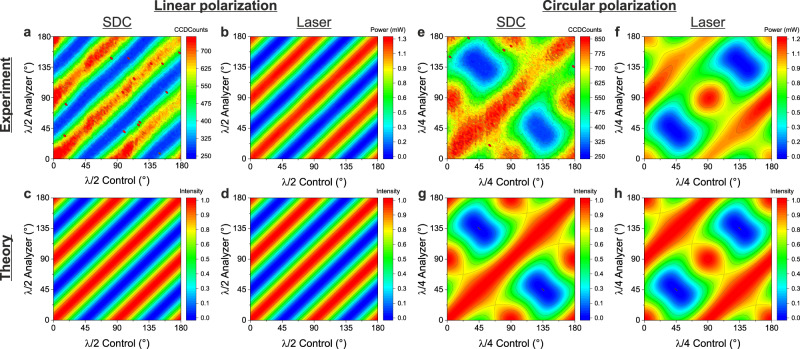
Polarization control of SDC emission. Intensity of SDC and backscattered control-laser in dependence of angle of the control and analyzer waveplates (**a**–**d** linear polarization, **e**–**h** circular polarization) The top row shows experimental results and the bottom row shows corresponding simulations.

In the first case we used a half-wave plate to rotate the orientation of the linearly polarized control-laser and we analyzed the resulting SDC-emission with a second half-wave plate and a polarizer in the detection path (see Supplementary Figure 2). In Fig. 6a, b we show experimental data for the SDC-emission and the backscattered control-laser light. In Fig. 6c, d we show corresponding simulations. A comparison of the results of the SDC and the backscattered laser light clearly shows, that the orientation of the SDC polarization nicely follows the laser polarization. The experimental data shows very good agreement with the simulations.

In the other case we followed a similar strategy using quarter-wave plates to investigate also circular polarization (see Fig. 6e–h). Upon rotation of the quarter-wave plate the Stokes vector of the control-laser follows an eight-shaped trajectory on the Pointcaré-sphere (see Supplementary Note 6) generating a more complex pattern. Pure circular polarization is obtained at 45° and 135°. In these cases, we observe a clear complementary polarization behavior of control and SDC. This shows that $s=+1$ control photons lead to the emission of $s=-1$ SDC photons. All other points on the trajectory nicely follow the predictions of the theoretical model, as can be seen from the good qualitative agreement between the complicated patterns obtained from theory and experiment.

## Discussion

Our experimental and theoretical results demonstrate in an unambiguous way stimulated down-conversion in a single QD. This is manifested by the characteristic Stark-effect shift of the SDC-emission, which is a consequence of the conservation of energy. Furthermore, it is manifested by the observed polarization relations between the control-laser and the SDC emission. Conceptually, SDC lays the foundations for optical devices on the single quantum level. The down-conversion process works best in close spectral proximity of the $|X\rangle$ state. With a tuning range up to 0.5 meV, the down-converted emission is observable also far from the avoided crossing with the $|X\rangle$ state. With an exceptionally good agreement between theory and experiment, the theory provides additional insights that complement the available experimental evidence. This is true in particular for the appearance of the Mollow-triplet, that is not observable in the experiment, as a result of the required stray-light suppression. The appearance of this spectral feature underlines the relation between the SDC concept and the dressed state picture. Both models describe the same underlying physics but with a focus on different detuning regimes. In our work we advanced to larger detunings (up to 500 µeV), where the concept of SDC is justified and advantageous. Based on the spin properties of the involved states $|B\rangle$ and $|G\rangle$, the SDC model directly provides a set of selection rules for the polarization of the emitted photons, which we verified for the two important cases of linear and circular polarization. Overall, our results show that the SDC model provides an intuitive and powerful description of optically controllable single photon emission. For future applications we expect that the efficiency and tuning range of the SDC-process can be vastly improved by the employment of cavities with high Purcell-factor (see Supplementary Note 5—Power dependence of the SDC). This will enhance the SDC-emission and suppress the competing emission cascade via the $|X\rangle$ state^23^. Pulsed two-photon excitation allows for the deterministic preparation of the $|B\rangle$-state. Control over the timing and the spectral characteristics of the single photon emission can be gained by tailored control-laser pulses. Overall, our results open attractive pathways for the optical control of quantum emitters by nonlinear concepts.

## Methods

### Theoretical model

To model the optical excitation of the quantum dot system we include four electronic configurations—the ground state $|G\rangle$, two exciton fine structure states $|{X}_{H}\rangle$ and $|{X}_{V}\rangle$ as well as the biexciton $|B\rangle$ —with the Hamiltonian

4 $${H}_{0}={E}_{G}|G{\rangle}{\langle }G{|}+{E}_{H}{|}{X}_{H}{\rangle }{\langle }{X}_{H}{|}+{E}_{V}{|}{X}_{H}{\rangle}{\langle }{X}_{H}{|}+{E}_{B}{|}B{\rangle }{\langle}B{|}$$

We adjust the voltage dependent (bi-)exciton energies to the experimental data shown in Fig. 6. Corresponding to the experimental situation, two lasers couple adjoining electronic states with the selection rules

5 $${H}_{int}=\mathop{\sum} \limits_{{i}={H},{V}}(({|}G{\rangle}{\langle }{X}_{i}{|}{\varOmega }_{i}^{* }(t)+{|}{X}_{i}{\rangle }{\langle }B{|}{\varOmega }_{i}^{* }(t))+{{\mbox{h.c.}}})$$

with ${\varOmega }_{H}$ and ${\varOmega }_{V}$ being the amplitude of the TPE and control-laser, respectively. Here, we assume cw-excitation

6 $${\varOmega }_{i}(t)=\frac{{\varOmega }_{i}^{0}}{2}{\exp }({i}{\omega }_{{{{{\rm{i}}}}}}{{{{{\rm{t}}}}}})$$

with ${\varOmega }_{H}^{0}=0.3\,{{{{{{\rm{m}}}}}}}{{{{{{\rm{eV}}}}}}}$ and the down-conversion laser with ${\varOmega }_{V}^{0}=0.198\,{{{{{{\rm{m}}}}}}}{{{{{{\rm{eV}}}}}}}$ (*E*_control_ close to *E*_*X*_) and ${\varOmega }_{V}^{0}=0.189\,{{{{{{\rm{m}}}}}}}{{{{{{\rm{eV}}}}}}}$ (*E*_control_ close to *E*_*XX*_), respectively, as determined experimentally. The pulse frequencies ${\omega }_{i}$ are chosen according to the experimental data in Fig. 3. For comparison with the measured spectra, the theoretical emission intensity^31^ is calculated with respect to the electronic quantum dot operator ${\sigma }_{V}={|G}\rangle \langle {X}_{V}|+|{X}_{V}\rangle \langle {B|}$ in the same linear mode as the control pulse^32^ as

7 $${S}_{V}(\omega )={\mathfrak{R}}{\int }_{0}^{{t}_{0}^{V}}{dt}{\int }_{0}^{{t}_{0}^{V}-t}d\tau {{\langle }}{\sigma }_{V}^{{{\dagger}} }(t){\sigma }_{V}(t+\tau ){{\rangle }}{e}^{i\omega \tau }{e}^{-\frac{\varGamma }{2}({t}_{0}^{V}-t)}{e}^{-\frac{\varGamma }{2}({t}_{0}^{V}-t-\tau )}$$

with spectral width $\hslash \varGamma =40\,\upmu {{{{{{\rm{eV}}}}}}}$ of the detector. The integrated emission intensity is limited by the spectral width of the detector and the time window is chosen to be ${t}_{0}^{V}$ = 480 ps. We evaluate the two-time expectation values using the quantum regression theorem^33^ and taking the trace with the density matrix of the quantum dot system ${\rho }_{s}$ which obeys^23,32^

8 $$\frac{\partial {\rho }_{s}}{\partial t} \,=	\, \frac{1}{i{{\hslash }}}[{H}_{0}+{H}_{{int}},{\rho }_{s}]-\frac{{\gamma }_{{pure}}}{2}\mathop{\sum} \limits_{\chi ,{\chi }^{{\prime} };\chi \ne {\chi }^{{\prime} }}|\chi \rangle \langle \chi |{\rho }_{s}|{\chi }^{{\prime} }\rangle \langle {\chi }^{{\prime} }|\\ 	+\frac{{\gamma }_{{rad}}}{2}\mathop{\sum} \limits_{i={X}_{H},{X}_{V}}\left({{{{{{\mathscr{L}}}}}}}_{|G\rangle \langle i|}+{{{{{{\mathscr{L}}}}}}}_{|i\rangle \langle B|}\right)({\rho }_{s})+\frac{{P}_{{{{{{{\rm{incoh}}}}}}}.}}{2}{{{{{{\mathscr{L}}}}}}}_{|B\rangle \langle G|}({\rho }_{s})$$

where $\chi ,{\chi }^{{\prime} }\in \{G,{X}_{H},{X}_{V},B\}$ and ${{{{{{\mathscr{L}}}}}}}_{\sigma }({\rho }_{s})=(2\sigma {\rho }_{s}{\sigma }^{{{\dagger}} }-{\sigma }^{{{\dagger}} }\sigma {\rho }_{s}-{\rho }_{s}{\sigma }^{{{\dagger}} }\sigma )$. We choose the pure dephasing rate $\hslash {\gamma }_{{{{{{{\rm{pure}}}}}}}}=4\,\upmu {{{{{{\rm{eV}}}}}}}$, a radiative decay $\hslash {\gamma }_{{rad}}=1\,\upmu {{{{{{\rm{eV}}}}}}}$, and an incoherent pump $\hslash {P}_{{{{{{{\rm{incoh}}}}}}}.}=5\,\upmu {{{{{{\rm{eV}}}}}}}$. In addition to the light pulse, the biexciton to ground state transition is pumped incoherently by the last term to account for biexciton generation even if the driving lasers are off-resonant to a (cascaded) two-photon excitation scheme. For efficient evaluation, the calculations are performed in the interaction picture such that the relatively large carrier frequencies in ${H}_{0}$ do not have to be treated explicitly numerically. We note that with increasing pure dephasing the intensity of the SDC emission is gradually reduced and pure dephasing as well as radiative decay set a lower limit for the achievable linewidth of the SDC emission as discussed in more detail by Breddermann et al.^24^.

### Spectroscopic setup

The sample is placed in a low temperature microscope setup, which is cooled down to T = 4.2 K (see Supplementary Fig. 1a). An aspheric lens with NA = 0.68 is used to focus the laser light onto the specimen and to collect its emission in a confocal geometry. As excitation sources we use two independently tunable continuous wave (cw) lasers, both fiber-coupled to the head of the microscope: An external cavity diode laser for the TPE and a Ti:Sapphire-laser in cw-mode as control-laser. The emission of both lasers is filtered with tunable band-pass filters with a bandwidth of 0.45 nm to suppress sideband emission (not shown in Supplementary Fig. 1a). The QD emission is collected with a single-mode optical fiber and sent to a spectrometer. In order to detect the QD emission, the stray light resulting from the two lasers must be suppressed. For the TPE laser, this is realized by a combination of a polarization suppression scheme based on the setup published by Kuhlmann et al.^34^ and a tunable notch-filter with a bandwidth of 0.7 nm in front of the spectrometer. Because of the selection rules, the control-laser cannot be suppressed by cross-polarization. Therefore, we use spectral filtering with two additional notch-filters. This setup finally allows for s-polarized excitation by the TPE laser, a p-polarized control-laser and detection of the p-polarized contribution of the QD emission.

For the measurements on polarization control the experimental setup needed to be modified. The polarization suppression for the TPE laser was removed from the system since the polarization beam splitter restricts both lasers to a fixed polarization. The straylight suppression is now achieved by two notch-filters per laser. A wave plate (λ/2 or λ/4) in the exciation path of the control-laser allows to vary its polarization during the experiments. It is referred to as “Control”. A corresponding waveplate in combination of a fixed polarizer in the detection path allows to analyze the QD emission and the backscattered laser light with regard to polarization. It is therefore referred to as “Analyzer”. In Supplementary Fig. 2 we show a sketch of this setup.

### Sample

In our experimental realization we use (In,Ga)As QDs grown by molecular beam epitaxy (MBE), which are embedded in a GaAs p-i-n photodiode (see Supplementary Fig. 1c) to allow for Stark tuning of the transition energies. The QDs are placed in the center of the 320 nm thick intrinsic layer. The areal density of the embedded (In,Ga)As-QDs is in the range of 1 QD/µm², low enough to allow for single QD spectroscopy. The buried n-GaAs region has a thickness of 110 nm and a Silicon doping concentration of 2 × 10^18^ cm^−3^. The Carbon doped p-GaAs region consist of a 95 nm thick layer with a doping concentration of 4 × 10^18^ cm^−3^, followed by a 15 nm thick surface layer with a doping concentration of 2 × 10^19^ cm^−3^. From this material, large area quantum dot photodiodes (550  × 700 μm^2^) are processed. On top of those, super-hemispherical solid immersion lenses (diameter 500 μm) are placed to enhance the detection efficiency and light-matter interaction (see Supplementary Fig. 1b).

### Theoretical model for the polarization control

For the theoretical description of the experiments we employed a theoretical model based on the Mueller calculus^35^. Within this formalism the effect of the SDC-process (inverting the sign of *s*_3_) is described by:

9 $${M}_{{{{{{{\rm{SDC}}}}}}}}=\left(\begin{array}{cccc}1 & 0 & 0 & 0\\ 0 & 1 & 0 & 0\\ 0 & 0 & 1 & 0\\ 0 & 0 & 0 & -1\end{array}\right)$$

We then start with the Stokes vector of linear horizontal polarization S_H_ and apply a series of Mueller matrices to this vector that describe the effect of the waveplates (${M}_{\lambda /x}$, with λ/x = λ/2 or λ/4), the SDC process (${M}_{{{{{{\rm{SDC}}}}}}}$) and the horizontal polarizer (${M}_{{{{{{\rm{H}}}}}}-{{{{{\rm{Pol}}}}}}}$) in the detection path to map the intensity of the SDC emission in dependence of the two waveplate angles ${\theta }_{1}$and ${\theta }_{2}$:

10 $${S}_{{{{{{{\rm{out}}}}}}},{{{{{{\rm{SDC}}}}}}}}={M}_{{{{{{\rm{H}}}}}}-{{{{{{\rm{Pol}}}}}}}}\cdot {M}_{\lambda /x}\left({\theta }_{2}\right)\cdot {M}_{{{{{{{\rm{SDC}}}}}}}}\cdot {M}_{\lambda /x}\left({\theta }_{1}\right)\cdot {S}_{{{{{{\rm{H}}}}}}}$$

For the intensity of the backscattered laser light we perform an equivalent calculation but without the matrix for the SDC process:

11 $${S}_{{{{{{{\rm{out}}}}}}},{{{{{{\rm{Laser}}}}}}}}={M}_{{{{{{\rm{H}}}}}}-{{{{{{\rm{Pol}}}}}}}}\cdot {M}_{\lambda /x}\left({\theta }_{2}\right)\cdot {M}_{\lambda /x}\left({\theta }_{1}\right)\cdot {S}_{{{{{{\rm{H}}}}}}}$$

## Supplementary information

Supplementary information

## Data Availability

The original data generated in this study have been deposited in the zenodo database under accession code 10.5281/zenodo.6024228.

## Code availability

The code that was used to analyze the experimental data is available from the corresponding authors upon reasonable request.
