# Supplementary material for: Nonlinear down-conversion in a single quantum dot
Source: Nat Commun. 2022 Mar 16;13:1387. doi: 10.1038/s41467-022-28993-3 (PMC8927346; doi:10.1038/s41467-022-28993-3)
Supplement: Supplementary file 1 — Supplementary information [file 41467_2022_28993_MOESM1_ESM.pdf]

Nonlinear down-conversion in a single quantum dot – Supplementary Material

B. Jonas[1,2,3], D. Heinze[1,2,3], E. Schöll[1,2,3], P. Kallert[1,2,3], T. Langer[1,2,3], S. Krehs[1,2,3], A. Widhalm [1,2,3], K. D. Jöns [1,2,3], D. Reuter [1,2,3], S. Schumacher [1,2,3,4,*], and A. Zrenner [1,2,3,†]

[1] Paderborn University, Physics Department, Warburger Straße 100, 33098 Paderborn, Germany
[2] Paderborn University, Center for Optoelectronics and Photonics Paderborn (CeOPP), Warburger Straße 100, 33098 Paderborn, Germany
[3] Paderborn University, Institute for Photonic Quantum Systems (PhoQS), Warburger Straße 100, 33098 Paderborn, Germany
[4] Wyant College of Optical Sciences, University of Arizona, Tucson, Arizona 85721, USA

[*]stefan.schumacher@upb.de
[†]artur.zrenner@upb.de

Supplementary Note 1 - Sketches of the experimental setups

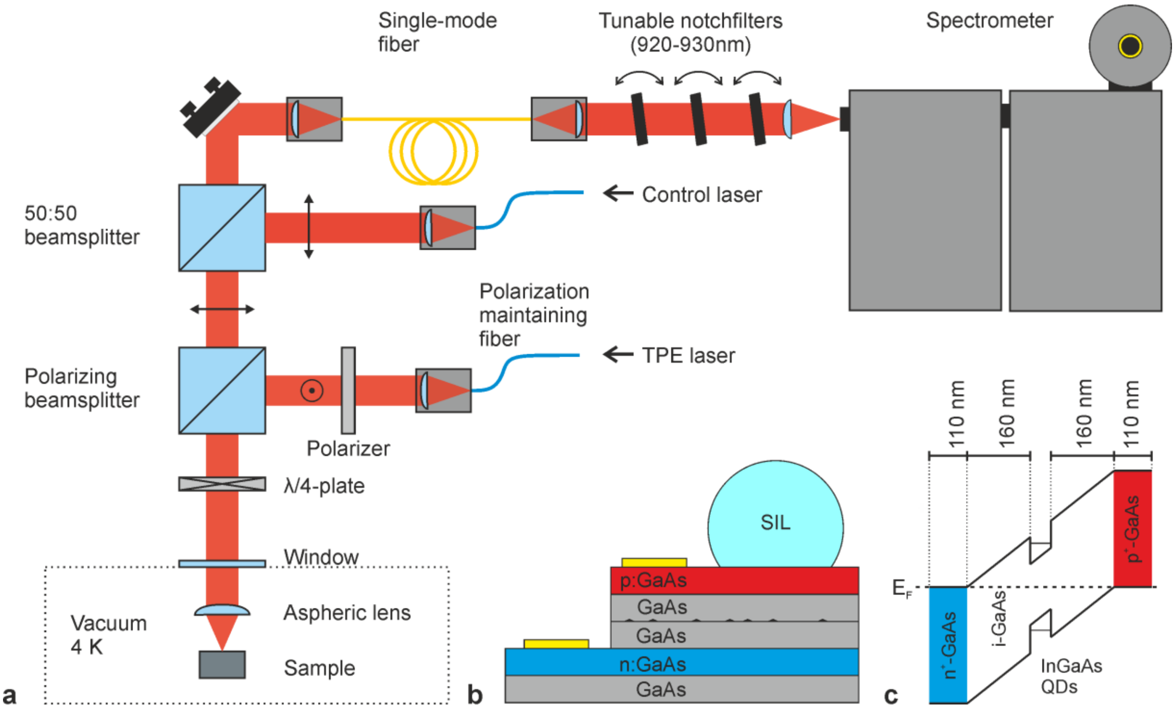

Supplementary Figure 1 | Experimental setup and sample structure. a, Optical setup for the down-conversion experiment (see also text). **b,** p-i-n QD mesa-diode with attached solid-immersion lens (SIL). **c,** Band diagram of the MBE-grown p-i-n structure with indicated thickness of the individual layers.

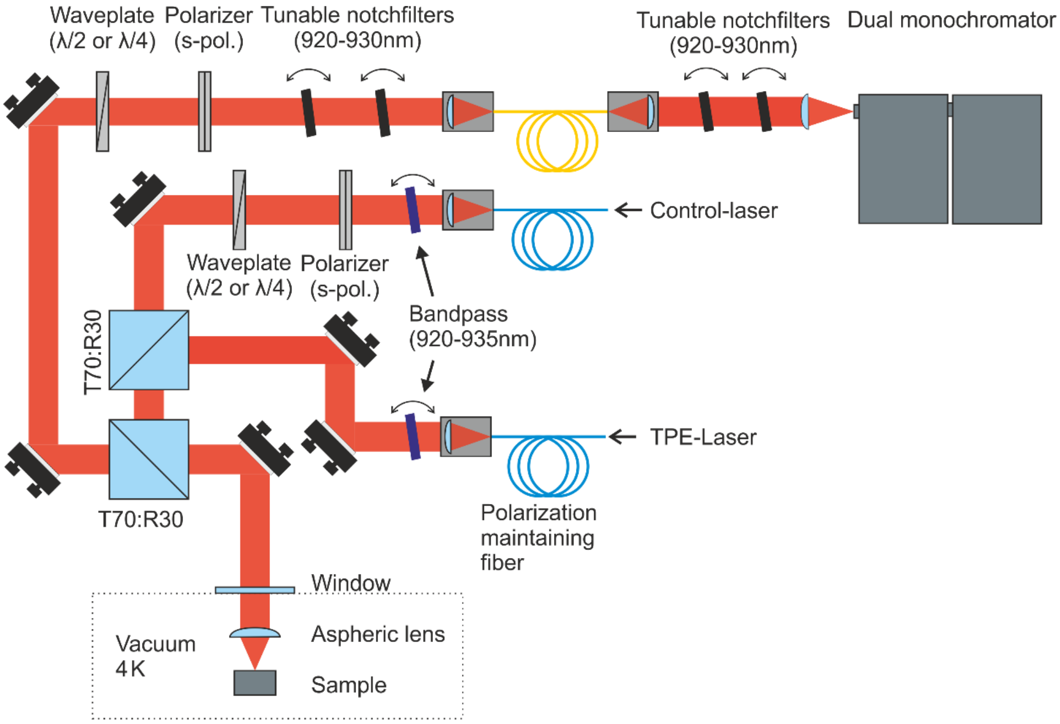

Supplementary Figure 2 | Experimental setup for the polarization control. The Setup relies completely on spectral filtering for straylight suppression. The polarization of the control-laser can be manipulated by a waveplate (Control) and the resulting QD emission can be analyzed by a corresponding waveplate in the detection path (Analyzer).

Supplementary Note 2 - Phonon-assisted two-photon excitation

The starting point is the initial state preparation for the optical down-conversion experiment illustrated in Fig. 1. Biexciton preparation can be achieved with a variety of excitation schemes[1–3]. In this work we use phonon-assisted two-photon excitation. This process has the advantage that it is very robust against detuning and changes of the laser power[4,5]. Applying a bias voltage (V_{Bias}) to the diode structure tunes the emission energies of the QD due to the Stark effect. Experimental data for a bias voltage controlled tuning range of about 2 meV is displayed in Fig. S3. The TPE laser is tuned to 1341.17 meV and set to an excitation power of 12 mW to achieve saturated excitation conditions[4,24,25].

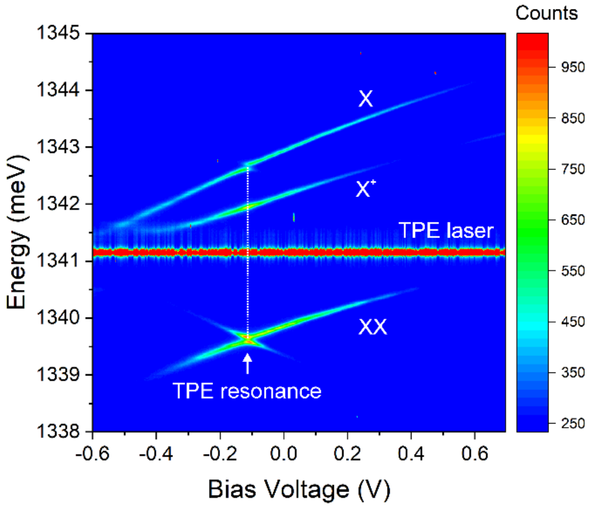

Supplementary Figure 3 | Two-photon excitation of the biexciton. Bias voltage dependent results for the phonon assisted two-photon excitation of the biexciton by a TPE laser without control-laser. The sequential decay of the biexciton leads to the XX- and X-emission lines. The emission of the positive trion-line X^+ appears statistically in the biased diode structure. At the TPE-resonance, the avoided crossings of the XX- and X-lines are caused by the dressing of the $|B\rangle$- and $|G\rangle$-states (T_{int}=1 s).

Due to the polarization suppression scheme we only detect light with a polarization perpendicular to the TPE laser. The measurement shows the emission of the XX-/X-cascade and an emission of the positive trion (X^+), which statistically appears in the biased diode structure. At V_{Bias} = -0.12 V the laser is in resonance with the direct two-photon transition and we achieve direct two-photon excitation without the need of phonons. As a result of the high excitation power, an avoided crossing in the X- and XX-line is observed, which is the result of the dressing of the $|B\rangle$- and $|G\rangle$-states[6–8].

The phonon assisted TPE provides the preparation of the initial state for our down-conversion experiment. For this we introduce a control-laser, which is tuned close to the energy of either the XX- or X-line. Due to the selection rules for the down-conversion process, the emitted photons have the same linear polarization as the control-laser. In our setup with polarization suppression for the TPE-laser, the polarization of the control-laser has to be perpendicular to the TPE laser. Therefore, this laser can only be suppressed by spectral filtering, which we realize with two tunable notch-filters.

Supplementary Note 3 - SDC intensity versus detuning

We have analyzed the data presented in Fig. 3 with regard to the dependency between the SDC intensity and its detuning relative to the undisturbed X- ($E-E_X$) or XX-line ($E-E_{XX}$). For the experimental data this was done by fitting the obtained spectra with Lorentzian functions in order to extract the peak height. In order to account for changes of the overall intensity of the QD emission upon changing the diode voltage, the obtained peak height was normalized to the sum of SDC and X/XX. The energy of the undisturbed emission was obtained from an analysis of the Stark-effect in Fig. S1. The theoretical data was treated in a similar fashion. Due to the absence of experimental noise the height of the peaks could be directly taken from the data without fitting. Fig. S4 shows the results of this analysis. We find that the decline of the SDC intensity with increasing detuning from the undisturbed QD emission is well-described by a Lorentzian function. In the case were the SDC appears close to the XX-line, we find a very good agreement between experiment and theory. In the other case of the SDC close to the X-line, the experimental data shows a clear asymmetry with stronger emission on the low energy side. This asymmetry is not observable in the theoretical data. The reason behind this asymmetry is still unclear. A possible explanation are phonon-processes, which are not included in the theoretical model. As a result of this asymmetry the SDC works best in the low energy branch close to the X-line.

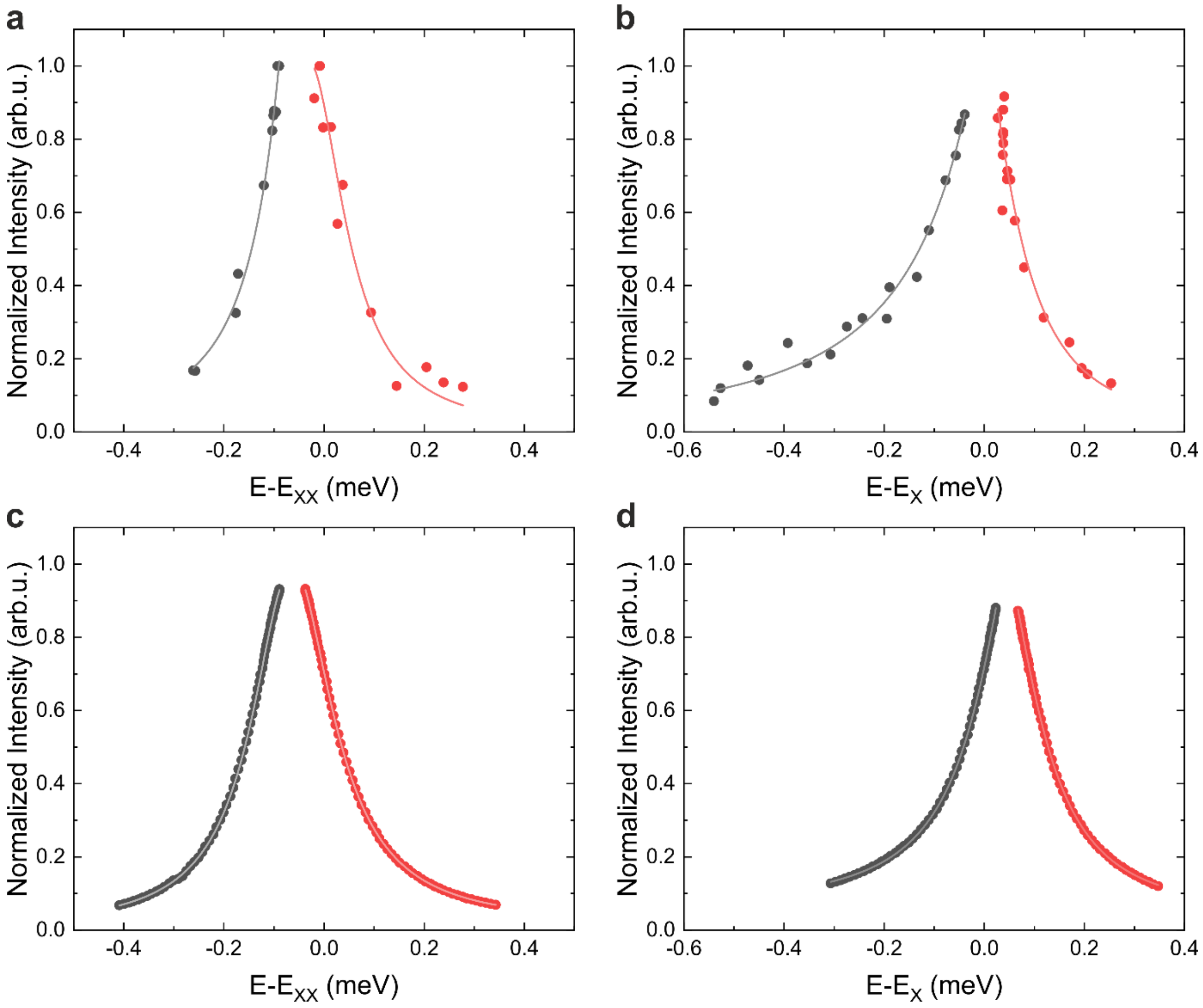

Supplementary Figure 4| Detuning dependence of SDC intensity. The intensity of the SDC emission was extracted from the experimental data displayed in Fig. 3 a,b and plotted versus its detunig from the undisturbed X- (**a**) or XX-emission (**b**). **c, d** Corresponding simulation data. The low energy side (black) and the high energy side (red) were fitted with independent Lorentzian functions.

Supplementary Note 4 - Determination of the Rabi energy

To determine the control-laser-induced Rabi frequency from the experimental data shown in Fig. 3 a and b, we examined the splitting between the two lines of avoided crossing of the SDC emission with the X and XX lines (see Fig. S5). We find that the data is reasonably well described by $\Delta_{Splitting} = \sqrt{\Omega_0^2 + \Delta_{Control}}$. Especially around the minimum we observe some deviation between the data and the fit. This caused deviations between the experimental and theoretical data when the $\hbar\Omega_0$ obtained from the fit was used as an input for the simulation. We therefore used the data point with the smallest splitting to determine the Rabi energy, which delivered a better agreement between experiment and simulation.

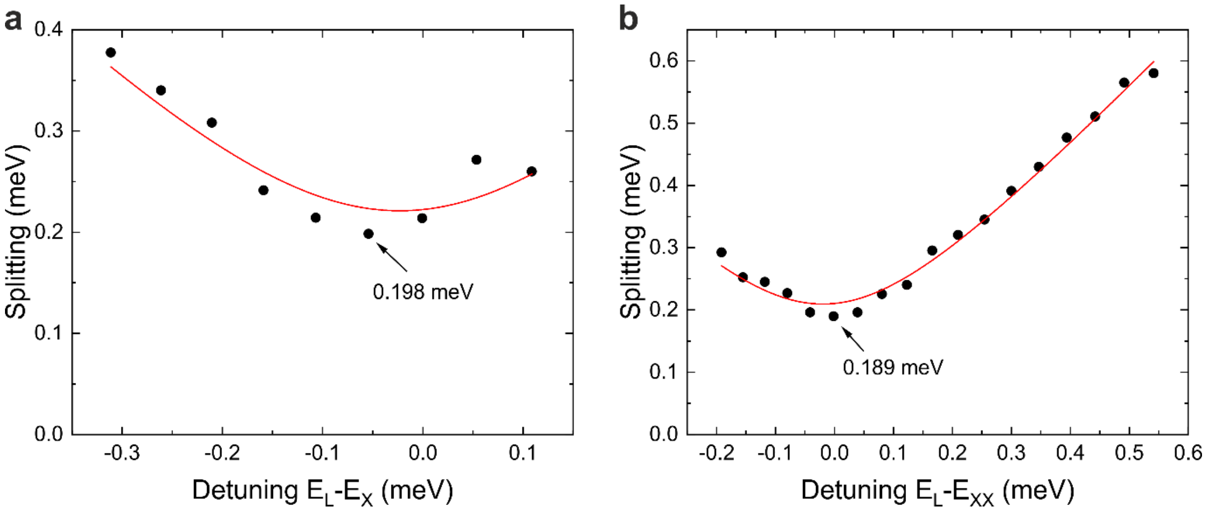

Supplementary Figure 5| Determination of the Rabi energy. The splitting between the SDC and the XX-line (**a**) or the X-line (**b**) is plottet versus the detuning of the control-laser relative to the X- (**a**) and XX-line (**b**). The data was obtained by fitting a subsample of the spectra displayed in Fig. 3 a,b with Lorentzian functions and extracting the Stark-effect from the data displayed in Fig. S3.

Supplementary Note 5 – Power dependence of the SDC

We furthermore investigated the relation between the intensity of the SDC emission I_{SDC} and the power of the control-laser. Fig. S6 shows an experimental series (a) and a corresponding simulation (b) for different control-laser powers with a control-laser detuning of +0.33 meV relative to the XX emission. The experimental data shows a clear linear behavior for low control power. Towards higher power the data shows slight indication for a saturation behavior. Due to experimental restrictions, it was not possible to further increase the control power, but our power dependent theoretical data clearly shows saturation under strong excitation. The range of available experimental data is indicated in Fig. S6 b. Both the experimental and theoretical data are well-described by a fitting function that models the nonlinear saturation of a two-level system[31,32]:

$$I_{SDC}(\tilde{P}) = I_{sat} \cdot \frac{\tilde{P}}{\tilde{P}+1} \tag{1}$$

Here, $\tilde{P}$ is a normalized control power which is proportional to Ω_0^2 and the power of the control-laser. The dependence $\sim\Omega_0^2$ is also found theoretically for a quantum dot inside a cavity.[9]

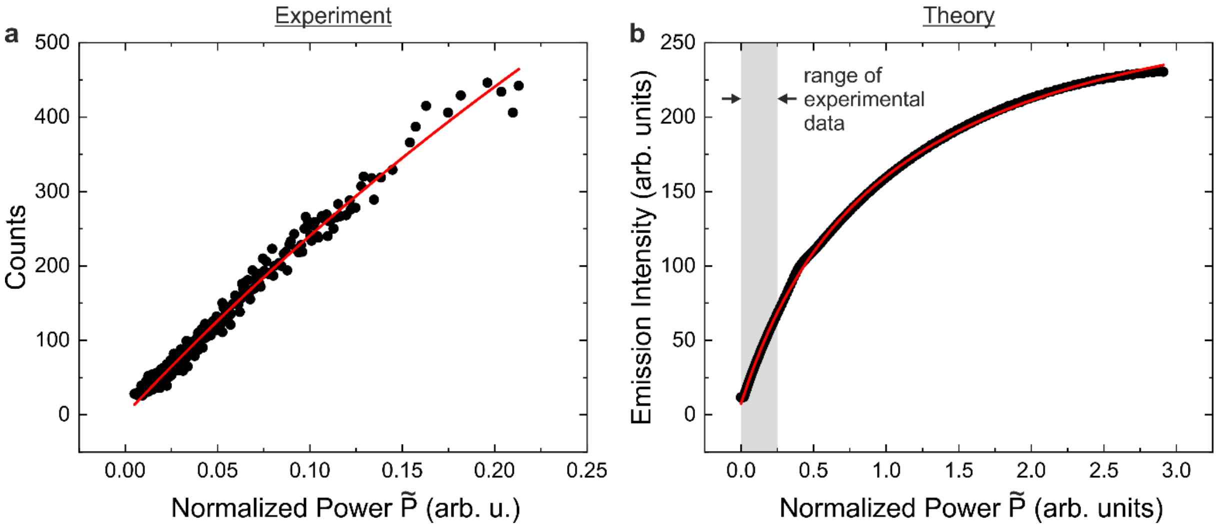

Supplementary Figure 6 | Intensity of the SDC emission for different control powers. a, Experimental data $\tilde{P}_{Exp} = 0.06266\ (mW)^{-1} \cdot P$. **b,** Theoretical data $\tilde{P}_{Theo} = 4.54869\ (meV)^{-2} \cdot (\hbar\Omega_0)^2$

We would like to briefly discuss how the SDC intensity depends on system parameters in the presence of an optical cavity mode. In that case the SDC intensity I_{SDC} scales like $I_{SDC}\sim g^2$, with g being the coupling strength of optical quantum dot transitions to the cavity mode, as discussed in detail in Ref. 9. SDC photon emission into a cavity mode with finite spectral width, the optical control of the SDC emission frequency can only be efficiently performed inside the cavity line with reduced SDC photon emission probability when not near resonant with the cavity, as discussed by Heinze et al.[10]. Consequently, the use of a high-Q or spectrally narrow cavity limits the possible spectral control, however, the optical control of polarization remains unaffected for cavities with degenerate polarization states.

Supplementary Note 6 - Visualization of the influence of wave plates

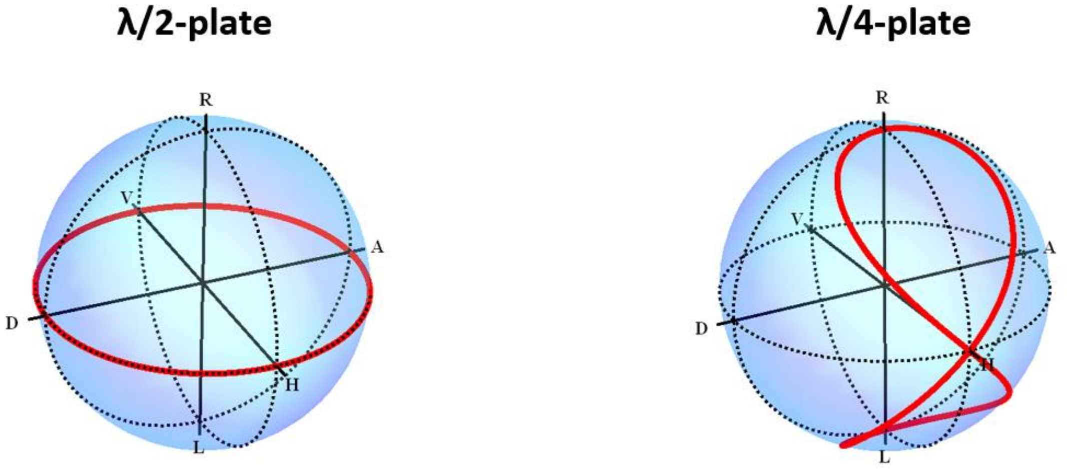

Supplementary Figure 7| Visualization of waveplates. Trajectory of the Stokes-vector on the Pointcaré-sphere upon rotation of a λ/2-plate (left) and a λ/4-plate (right) for horizontal Input-polarization.

References

1. Stufler, S. *et al.* Two-photon Rabi oscillations in a single $In_xGa_{1-x}As$ / GaAs quantum dot. *Phys. Rev. B* **73**; 10.1103/PhysRevB.73.125304 (2006).

2. Boyle, S. J., Ramsay, A. J., Fox, A. M. & Skolnick, M. S. Two-color two-photon Rabi oscillation of biexciton in single InAs/GaAs quantum dot. *Physica E: Low-dimensional Systems and Nanostructures* **42,** 2485–2488; 10.1016/j.physe.2009.11.008 (2010).

3. Chen, G. *et al.* Biexciton Quantum Coherence in a Single Quantum Dot. *Phys. Rev. Lett.* **88**; 10.1103/PhysRevLett.88.117901 (2002).

4. Glässl, M., Barth, A. M. & Axt, V. M. Proposed robust and high-fidelity preparation of excitons and biexcitons in semiconductor quantum dots making active use of phonons. *Phys. Rev. Lett* **110,** 147401; 10.1103/PhysRevLett.110.147401 (2013).

5. Ardelt, P.-L. *et al.* Dissipative preparation of the exciton and biexciton in self-assembled quantum dots on picosecond time scales. *Phys. Rev. B* **90,** 1065; 10.1103/PhysRevB.90.241404 (2014).

6. Jundt, G., Robledo, L., Högele, A., Fält, S. & Imamoğlu, A. Observation of Dressed Excitonic States in a Single Quantum Dot. *Phys. Rev. Lett* **100**; 10.1103/PhysRevLett.100.177401 (2008).

7. Hargart, F. *et al.* Cavity-enhanced simultaneous dressing of quantum dot exciton and biexciton states. *Phys. Rev. B* **93,** 681; 10.1103/PhysRevB.93.115308 (2016).

8. Bounouar, S. *et al.* Path-Controlled Time Reordering of Paired Photons in a Dressed Three-Level Cascade. *Phys. Rev. Lett* **118,** 233601; 10.1103/PhysRevLett.118.233601 (2017).

9. Breddermann, D., Praschan, T., Heinze, D., Binder, R. & Schumacher, S. Microscopic theory of cavity-enhanced single-photon emission from optical two-photon Raman processes. *Phys. Rev. B* **97**; 10.1103/PhysRevB.97.125303 (2018).

10. Heinze, D., Breddermann, D., Zrenner, A. & Schumacher, S. A quantum dot single-photon source with on-the-fly all-optical polarization control and timed emission. *Nature communications* **6,** 8473; 10.1038/ncomms9473 (2015).
